# DOPC Liposomal Formulation of Antimicrobial Peptide LL17-32 with Reduced Cytotoxicity: A Promising Carrier Against *Porphyromonas gingivalis*

**DOI:** 10.3390/pharmaceutics17111424

**Published:** 2025-11-04

**Authors:** Jinyang Han, Josephine L. Meade, Francisco M. Goycoolea

**Affiliations:** 1 School of Food Science and Nutrition, University of Leeds, Leeds LS2 9JT, UK; kristinajinyang@gmail.com; 2 School of Dentistry, Oral Biology, University of Leeds, Leeds LS2 9JT, UK; 3 Department of Cell Biology and Histology, Faculty of Biology, Universidad de Murcia, 30100 Murcia, Spain

**Keywords:** antimicrobial peptide (AMP), LL17-32, liposome, *Porphyromonas gingivalis*, periodontal diseases, dioleoyl-phosphatidylcholine (DOPC), soya lecithin (SL)

## Abstract

**Background/Objectives:** The rapid emergence of antibiotic-resistant oral pathogens has rendered many conventional therapies increasingly ineffective. Antimicrobial peptides (AMPs) have emerged as a promising therapeutic alternative due to their unique mechanisms of action and low propensity for inducing resistance. The delivery of novel therapeutic AMPs against oral cavity bacterial infections requires effective pharmaceutical dosage formulations. This study investigated the potential of two liposomal formulations for the association and delivery of the antimicrobial peptide (AMP) LL17-32 against the dental bacterial pathogen *Porphyromonas gingivalis*. **Methods:** Liposomes composed of either negatively charged soya lecithin (SL) or neutrally charged dioleoyl-phosphatidylcholine (DOPC) phospholipids were formulated and characterized based on their hydrodynamic size distribution, ζ-potential, morphology, membrane fluidity, peptide association efficiency, stability and release of peptide in vitro under physiological conditions. The characterization of their biological activity included efficiency of bacterial killing, bacterial adherence, and mammalian cell cytotoxicity using human gingival keratinocyte (TIGK) cells. **Results:** Both liposomal formulations exhibited spherical morphology with hydrodynamic diameters smaller than ~170 nm and demonstrated good colloidal stability. LL17-32 showed high association efficiency with both liposomal membranes, with no detectable LL17-32 in vitro release. In biological assays, peptide-loaded DOPC liposomes exhibited dose-dependent bactericidal activity against *P. gingivalis*, whereas SL liposomes significantly attenuated the bactericidal effect of LL17-32. Both formulations displayed reduced cytotoxicity toward human gingival keratinocyte (TIGK) cells versus free peptide. **Conclusions:** These findings suggest that DOPC liposomes represent a promising delivery system for LL17-32 by adhering to *P. gingivalis* and exhibiting minimal cytotoxicity to mammalian cells. This study emphasises the critical role of lipid charge in designing AMP delivery systems for antibacterial applications, while it additionally demonstrates the utility of flow cytometry as a quantitative tool to assess liposome–bacteria association.

## 1. Introduction

Periodontal diseases (PDs) generally refer to an inflammatory pathologic state of the periodontium. Depending on disease progression, these may involve only the gingival tissues or extend to deeper components such as the periodontal ligament, cementum, and alveolar bone [1]. PDs have been reported to be associated with diabetes, cardiovascular diseases, adverse pregnancy outcomes, respiratory diseases, Alzheimer’s disease, rheumatoid arthritis, pancreatic cancer, and chronic kidney disease [2,3,4,5,6,7]. More than 50 billion bacteria, over 700 species, as well as viruses and fungi, colonize the oral cavity [8]. This stable community known as the climax community develops a set of dynamic processes involving complex intra- and interspecies signalling and host–microbe interactions. Many different factors can contribute to this finely balanced community being disrupted, including poor oral hygiene, dietary habits, smoking, gingival inflammation, genetic difference, and dysfunction of the salivary glands [9]. For this reason, if an imbalance of the resident oral microbiota occurs, known generically as dysbiosis, inflammatory PD can become established, resulting in the subsequent propagation of potentially pathogenic microorganisms [10].

*Porphyromonas gingivalis*, a keystone pathogen in chronic periodontitis, is a non-motile, saccharolytic, obligatory anaerobic Gram-negative bacterium, which can form black-pigmented colonies on blood agar. It has an absolute requirement for iron and relies on the fermentation of amino acids as a source of nitrogen and carbon. It has been reported that a dual lifestyle is exhibited by *P. gingivalis* in periodontal sites, living both within the subgingival sulcus dental plaque (biofilm) and intracellularly within gingival host cells [11]. In a biofilm, *P. gingivalis*, as a secondary colonizer of periodontal biofilm, often adheres to primary colonizers such as *Streptococci* and *Actinomyces*. It is suggested that the incorporation of *P. gingivalis* into a network of interconnected microbes elicits the full range of its pathogenicity [12]. There has been evidence of the presence of *P. gingivalis* within epithelial cells collected from periodontal pockets, gingival crevices, and buccal mucosa from patients with periodontitis as well as healthy volunteers [13]. By entering these cells, *P. gingivalis* can pass through the epithelial barrier into deeper tissues and enter gingival fibroblasts [14] and osteoblasts [15], thus resulting in the disruption of fundamental cellular functions like migration and proliferation [16]. These intracellular locations are considered advantageous for escape from host immune surveillance and antibiotic pressure, leading to intracellular persistence, multiplication, and dissemination to adjacent tissues [17,18,19]. *P. gingivalis* actively promotes a dysbiotic biofilm community and chronic inflammatory environment that support its survival and persistence [2,20]. Thus, a treatment which targets this pathogen could simultaneously affect the entire biofilm and suppress inflammation and disease development.

Numerous strategies have been explored to prevent PD by targeting oral bacteria, offering a potential avenue for novel therapeutics. Among these strategies, antimicrobial peptides (AMPs) have emerged as promising candidates due to their broad-spectrum activity against pathogenic microorganisms and their relatively lower propensity for the emergence of antibiotic-resistant strains. While certain AMPs have demonstrated strong antibacterial efficacy in vitro, some limitations hinder their clinical utility. These drawbacks include adverse effects on eukaryotic cells such as neurotoxicity, haemolysis, and nephrotoxicity. Additionally, AMPs can be susceptible to degradation by bacterial proteases and exhibit low bioavailability, and their pharmacokinetic profile remains inadequately understood [21]. To address these challenges, research has explored the use of liposomal and polymeric nanoparticle formulations as delivery vehicles for AMPs. Such approaches aim to mitigate the cytotoxicity, enable sustained release, protect against degradation and enhance the bactericidal efficiency of AMPs. However, it is important to note that this field is still in its nascent stages, and the underlying mechanisms are yet to be fully elucidated.

To date, the reported AMPs showing antimicrobial or/and antibiofilm activity against *P. gingivalis* include beta-defensins [22], human cathelicidin (LL-37) [23] and cathelicidin-related AMPs [24,25], lactoferrin derivatives [26], Nal-P-133 [27], Pac-525 [28], ZXR-2 [29], SspB Adherence Region (BAR) peptide mimetics [30], peptide gallic acid-polyphemusin I [31], PD7 [32], DGL 13K [33], and plant fungus pathogen *Inonotus hispidus* peptides [34]. Depending on the AMP amino acid sequence, their antimicrobial activities and mechanisms of action are dependent on their membrane-disrupting activity, targeting intracellular molecules such as enzymes and nucleic acids, and/or through interfering with the expression of virulence factors.

In a preceding paper [35], we documented the formulation of different types of chitosan-coated liposomes loaded with the antimicrobial peptide LL17-32 (FKRIVQRIKDFLRNLV), a fragment of the human cathelicidin LL-37, and examined their biophysical and antimicrobial properties. The chitosan coating imparted a strong positive surface charge, resulting in high peptide association efficiency and apparent antibacterial activity. However, subsequent analysis revealed that the antimicrobial effect was mainly attributable to the chitosan coating rather than the peptide itself, and that chitosan also reduced liposomal stability under physiological conditions. These findings motivated the current study, which employs the same peptide, LL17-32, as a model antimicrobial agent but focuses on uncoated lipid systems with different intrinsic charges—negatively charged soybean lecithin (SL) and neutral dioleoylphosphatidylcholine (DOPC)—to better isolate the role of lipid composition in peptide association, stability, and antibacterial performance.

LL17-32 was retained as the model peptide due to its physicochemically and biologically enhanced performance. Namely, LL17-32’s primary structure, generated by the removal of non-antimicrobial activity (1–12 residues) and residues (13–16 and 33–37 residues) not requisite to micelle-binding from LL-37, results in improved activity and efficacy. Moreover, this small peptide exhibits a notably high charge density. Specifically, under physiological pH conditions, six out of its sixteen amino acid residues carry electrical charges. Among these, two lysine and three arginine amino acids contribute to a total of five positive charges, while a single aspartic acid residue contributes with one negative charge. Consequently, the net charge of the peptide at physiological pH stands at ~+4. The peptide’s primary structure further reveals distinctive distribution patterns of its side chains. Residues F17, I20, V21, I24, F27, L28, L31, and V32 are situated on the peptide’s hydrophobic surface, whereas residues K18, R19, Q22, R23, D26, and R29 reside on the hydrophilic region. This structural arrangement is well-suited for targeting bacterial membranes characterized by negative charges [36]. Accordingly, in the present work, we formulated SL and DOPC liposomes, either loaded or unloaded with the LL17-32 peptide. We examined their biophysical and structural properties (size, ζ-potential, morphology, and membrane fluidity), colloidal stability, peptide association efficiency, and in vitro release. These studies were complemented by biological assays evaluating the antibacterial efficacy of both formulations against *P. gingivalis* and their interactions with bacterial cells. Finally, the cytotoxicity of these formulations was assessed using human telomerase-immortalized gingival keratinocytes (TIGK).

## 2. Materials and Methods

### 2.1. Materials

We purchased 18:1 (Δ9-Cis), PC 1, 2-dioleoyl-*sn*-glycero-3-phosphocholine (DOPC) (CAS: 4235-95-4) from Avanti Polar Lipids (Birmingham, AL, USA). Soybean lecithin (SL) was purchased from VWR Chemicals (CAS: 8030-76-0, Leuven, Belgium). Peptide LL17-32 was purchased from CPC Scientific Inc. (San Jose, CA, USA). Cholesterol (≥98%) (CAS: 57-88-5) was purchased from Sigma Aldrich (St. Louis, MO, USA). Laurdan (CAS: 74515-25-6) was purchased from Cayman Chemist (Ann Arbor, MI, USA). 1,1′-dioctadecyl-3,3,3′,3′-tetramethylindodicarbocyanine perchlorate (CAS: 127274-91-3) (DiD) was purchased from Tocris Bio-Techne Ltd. (Bristol, UK).

*P. gingivalis* W83 strain and hTERT TIGKs (human telomerase immortalized keratinocytes) were purchased from the American Type Culture Collection (ATCC, Manassas, VA, USA). Brain heart infusion broth was purchased from EO Labs (Bonnybridge, UK). Blood Agar was purchased from Sigma Aldrich (St. Louis, MO, USA). Horse Blood was purchased from Thermo Fisher Scientific (London, UK). Fluorescein isothiocyanate isomer I (FITC) (CAS: 3326-32-7) was purchased from Fluorochem (Derbyshire, UK). Cytotoxicity Det. Kit (LDH) and MTT (3-(4, 5-dimethylthiazol-2-yl)-2, 5-diphenyltetrazolium bromide) were purchased from Sigma Aldrich (St. Louis, MO, USA). DermaLife Basal Medium was purchased from Lifeline Cell Technology (San Diego, CA, USA). The other chemicals were of analytical reagent grade or higher.

### 2.2. Methods

#### 2.2.1. Preparation of LL17-32-Loaded/Unloaded Liposomes and Fluorescence Labelling

In this study, soybean lecithin (SL) liposomes and dioleoylphosphatidylcholine (DOPC) liposomes, either loaded with LL17-32 or left unloaded as controls, were prepared using the thin-film hydration method as reported in our previous study [35]. Briefly, SL/DOPC and cholesterol (12 mg/mL and 2 mg/mL, respectively) were dissolved in chloroform, evaporated under nitrogen, and further dried under vacuum overnight. The lipid film was hydrated with 10 mM NaCl (pH 4.5), forming large multilamellar vesicles (LMVs). The suspensions were then extruded through a 100 nm polycarbonate membrane using a block-holding mini-extruder (61000, Avanti Polar Lipids, Alabaster, AL, USA) to obtain large unilamellar vesicles (LUVs). To ensure efficient extrusion, the temperature was set above the phase transition temperature (T_m_) of each phospholipid: for DOPC liposomes, extrusion was carried out at room temperature, while for SL liposomes, extrusion was performed at >60 °C. For LL17-32-loaded liposomes, 600 µg/mL LL17-32 was added to the aqueous phase before hydration. Laurdan-labelled liposomes were prepared by adding Laurdan together with SL/DOPC and cholesterol in chloroform, to a final lipid concentration of 0.1 mg/mL and Laurdan concentration of 2 µM. For DiD perchlorate-labelled liposomes, the same method was used with an SL/DOPC-to-DiD mass ratio of 8:3.

#### 2.2.2. Particle Size and ζ-Potential Analysis

Particle size distribution and polydispersity (PDI) were assessed through the determination of apparent hydrodynamic radius $R_{h}$, which is associated with the diffusional dynamics of vesicles. For each liposome preparation, DLS-NIBS measurements were conducted immediately after sample preparation and then again after 33 days of storage at 4 °C. Liposome suspensions were diluted 100-fold before recording. The ζ-potential was measured using mixed laser Doppler velocimetry and phase analysis light scattering (M3–PALS) based on electrophoretic mobility (μe) values. Both type of measurements were conducted using a Malvern Zetasizer ZEN5600 (Malvern Instruments, Worcestershire, UK) appointed with a 4 mW He/Ne laser beam (λ = 633 nm).

#### 2.2.3. Transmission Electron Microscopy (TEM) Imaging

The morphological characteristics of both liposomes were analyzed using a JEM-1400Flash Electron Microscope (Jeol Ltd., Tokyo, Japan) through a negative staining method. To prepare specimens for this study, a carbon-coated copper grid was carefully handled with negative-pressure tweezers, and 7 μL of the sample was applied to its surface, allowing for a 30 s adsorption period. Excess liquid was removed by gently touching the grid’s edge with filter paper. Subsequently, a 1% uranyl acetate solution was applied as a staining agent for 10 s, following the same procedure. The grid was then left to air dry or gently dried under an incandescent lamp before examination under the electron microscope. The examination of these samples was carried out using TEM at the Biostructure Laboratory, University of Leeds, Leeds, UK.

#### 2.2.4. Determination of Association Efficacy (AE)

The association efficiency (AE) of LL17-32 in different liposomal formulations was assessed using the ultrafiltration HPLC method. To perform this analysis, samples were subjected to a 2-fold dilution and introduced into ultrafiltration centrifuge tubes (PALL Nanosep, FE4687 10K Pall Corporation, NY, USA) and centrifuged at 15,000 rpm for 20 min (Eppendorf 5424 R, Eppendorf AG, Hamburg, Germany Eppendorf AG, Hamburg, Germany), followed by 1 min of vortexing and an additional 20 min centrifugation. The concentration of LL17-32 in the outer clear solution, representing the amount of free LL17-32, was determined using a reversed-phase HPLC method. A control sample of free LL17-32 solution was also tested.

For HPLC analysis, 20 µL of each sample was loaded onto an Agilent Eclipse XDB-C18 column (150 mm × 4.6 mm, 5 μm). The column was eluted using mobile phases A (0.1% *v*/*v* TFA in 10% acetonitrile in water) and B (0.85% *v*/*v* TFA in 90% acetonitrile in water) following this sequence: a linear gradient from 15% to 80% B over 13 min, holding at 80% B for 4 min, and then a linear gradient from 20% to 75% B over 2 min, all at a flow rate of 1 mL/min. Detection was performed at a wavelength of λ = 210 nm, and the column temperature was maintained at 60 °C. The retention time for LL17-32 was approximately 7.6 min. LL17-32 concentration was determined using a calibration curve standard (see Figure S1). HPLC analysis and data acquisition were conducted using the Nelson software version 5.x. The AE was calculated according to Equation (1).

(1) $$AE\left(\%\right)=\frac{{Total}_{LL17\text{-}32}-{Free}_{LL17\text{-}32}}{{Total}_{LL17\text{-}32}}\cdot 100$$

#### 2.2.5. In Vitro RTF Release Study

To evaluate the release of LL17-32 from SL/DOPC liposomes, a dialysis tube with a 10 kDa molecular weight cutoff, containing 300 μL of the formulations, was immersed in 30 mL of reduced transport fluid (RTF) at pH 7.1 [35]. RTF, characterized by its balanced mineral salt solution containing dithiothreitol, is also employed as an incubation medium in the peptide-induced bacteria-killing assay discussed in sections below. The RTF consisted of 10% EDTA, 20 mM NaCl, 4 mM sodium carbonate, 3 mM potassium dihydrogen phosphate, 1.2 mM ammonium sulphate, 1.5 mM dithiothreitol, and 2.5 mM dipotassium hydrogen phosphate. This system was maintained with continuous shaking at 80 rpm at 37 °C using a GRANT-JBA5 shaker (Grant Instruments, Cambridge, UK). At specific time intervals up to 24 h, samples were withdrawn from the mixture. To determine the concentration of released LL17-32, samples were concentrated using a Genevac 7EZ-2 plus apparatus (Genevac Ltd., Ipswich, UK). The released LL17-32 was then quantified by reversed-phase HPLC method as previously described in Section 2.2.4. Free LL17-32 was employed as a control, and the release rate was calculated using Equation (2).

(2) $$Release\;rate\left(\%\right)=\frac{{Released}_{LL17\text{-}32}}{{Total}_{LL17\text{-}32}}\cdot 100$$

#### 2.2.6. Physical Stability in RTF and Basal Culture Medium

The colloidal stability of liposomes (100 μL) in either RTF (pH 7.1) (900 μL) or basal culture medium (900 μL) was studied by monitoring the evolution of particle size distribution using DLS-NIBS described in Section 2.2.2. Measurements were taken up to 4 h in RTF at 37 °C (to reflect the bacterial study conditions) and up to 18 h in basal culture medium at 37 °C (to reflect the mammalian cell cytotoxicity study conditions) using the DLS-NIBS described in Section 2.2.2.

#### 2.2.7. Liposome Membrane Fluidity

To assess the impact of peptide LL17-32 on the fluidity of SL and DOPC membranes, we employed the fluorescent probe Laurdan (2-dimethylamino-6-lauroylnaphthalene) as a membrane probe [37]. Laurdan emits fluorescence at approximately λ = 490 nm in a liquid crystalline membrane, whereas in a gel phase membrane, it emits at λ = 440 nm. To quantify membrane fluidity, we calculated a generalized polarization (GP) by assessing the normalized intensity relationship. Laurdan-labelled liposomes were diluted before being placed in quartz cuvettes, and fluorescence emission spectra were acquired using an FP-8500 spectrofluorometer (JASCO Corporation, Tokyo, Japan) with an excitation wavelength of λ_ex_ = 340 nm and 2.5 nm slits. Spectra were recorded over the range of λ = 400 nm to 600 nm at temperatures of both 37 and 75 °C. GP was determined using Equation (3), where I440 and I490 represent the fluorescence emission intensity at λ_em_ = 440 nm (indicative of the gel phase) and λ_em_ = 490 nm (indicative of the liquid crystalline phase), respectively. Laurdan has also been widely employed to assess the lipid packing degree in the liquid crystalline phase (Lα-phase), providing a theoretical range of values from +1 (indicating the highest level of order) to −1 (representing the lowest level of order) [38].

(3) $$GP=\left(I_{440}-I_{490}\right)/\left(I_{440}+I_{490}\right)$$

#### 2.2.8. Bacteria-Killing Activity Against Porphyromonas gingivalis

The bacteria-killing activity of LL17-32 and liposomes against *P. gingivalis* was assessed using a method adapted from Curtis et al. [39]. Specifically, *P. gingivalis* W83 was cultured in brain heart infusion (BHI) broth and incubated in an anaerobic chamber (10% H_2_, 10% CO_2_, 80% N_2_) within the Whitley M55 Workstation (UK). The culture was allowed to reach the late logarithmic phase. Subsequently, the bacteria were washed twice in RTF at pH 7.1 and resuspended in RTF to achieve an optical density at 600 nm equivalent to 1 × 10^6^ colony-forming units per mL (CFUs/mL). For both free peptide LL17-32 and LL17-32-loaded/unloaded SL/DOPC liposomal formulations, a series of doubling dilutions was prepared in RTF within a 96-well plate, resulting in a final volume of 100 µL. Next, 100 µL of the bacterial suspension was added to achieve final concentrations ranging from 3.75 to 30 µg/mL. After incubation for either 30 min or 4 h, 20 µL aliquots from each well were diluted in 180 µL of RTF and subjected to tenfold serial dilutions. Then, 20 µL of the appropriate dilution was spread onto blood agar plates, which were incubated for 3–5 days to allow colony formation. Subsequently, viable bacterial colonies were counted, and viability rate was calculated using Equation (4):

(4) $$Viability\;rate(\%)=\frac{CFUs\;of\;the\;test\;sample}{CFUs\;of\;the\;control\left(untreated\right)}\cdot 100$$

#### 2.2.9. FITC Labelling of Porphyromonas gingivalis

By slightly altering the method reported previously [40], *P. gingivalis* strain W83 was grown to the late logarithmic phase. The concentration of bacteria was determined by spectrophotometry (at λ = 600 nm) according to a specific standard curve and confirmed retrospectively by counting viable cell colonies plated on blood agar for *P. gingivalis*. Bacteria were harvested by centrifugation at 8000× *g* for 5 min at 25 °C and washed once in phosphate-buffered saline (PBS, pH 7.4). *Bacterial pellets* (10^9^/mL) were resuspended in 0.5 M NaHCO_3_, pH 8.0, containing 0.05 mg/mL fluorescein isothiocyanate (FITC), incubated for 30 min at 37 °C in an anaerobic chamber (dark) with constant stirring, pelleted at 8000× *g* for 5 min, washed thrice with RTF to remove unbound FITC and suspended in RTF solution (pH 7.1). The optimal FITC concentration, providing homogenous labelling of intensity suitable for flow cytometric detection, was determined by a preliminary experiment (Supplementary information Figures S2 and S3).

#### 2.2.10. Treatment of LL17-32-Loaded Liposomes with *P. gingivalis*

The adherence of LL17-32-loaded SL/DOPC liposomes to *P. gingivalis* was also studied. FITC-labelled *P. gingivalis* W83 cells (600 µL) were incubated with non-DiD-labelled liposomes or series volume (1×, 2×, 4×) of DiD-labelled LL17-32-loaded liposomes at 10, 20, and 40 µL for 30 min at 37 °C in the anaerobic chamber. Mixtures were then centrifuged at 8000× *g* for 5 min at RT to remove excess unbound liposomes. The bacterial pellet with potentially bound liposomes was resuspended in RTF (pH 7.1) and diluted tenfold in RTF (pH 7.1) immediately before flow cytometry analysis.

#### 2.2.11. Flow Cytometric Analysis of LL17-32-Loaded Liposomes to *P. gingivalis*

**Figure 1:**
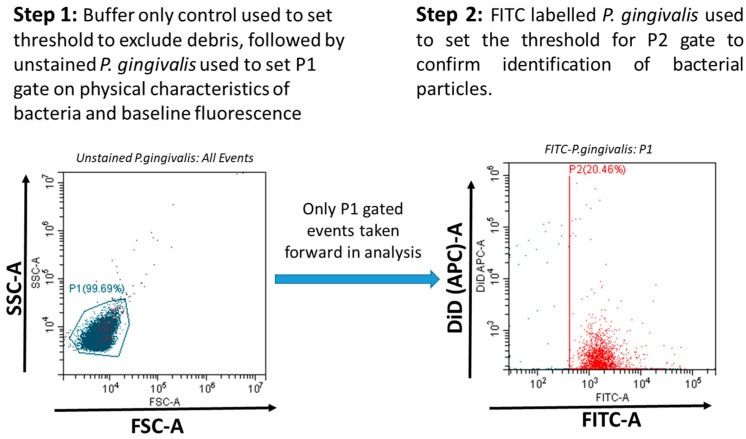
*P. gingivalis* was specifically identified within P1-gated events (indicated blue) by FITC fluorescence (with FITC-positive events being selected in the P2 gate, indicated red). The attachment of DiD-labelled LL17-32-loaded liposomes with *P. gingivalis* was measured by the increase in DiD fluorescence of FITC-positive bacteria (P2-gated).

The adherence of LL17-32-loaded SL/DOPC liposomes to *P. gingivalis* was analyzed using a CytoFLEXS flow cytometer (Beckman Coulter, Indianapolis, IN, USA) with the FITC excited by the blue 488 nm Argon laser and DiD perchlorate excited using the 638 nm red solid-state laser and detected in the allophycocyanin (APC) channel. Data was collected and analyzed using the CytExpert software package (Beckman Coulter, Indianapolis, IN, USA). Gating based on forward scatter (FSC) and side scatter (SSC) (proxy measurements indicating size and internal structural complexity) and comparison to an “assay buffer-only control” was used to exclude irrelevant debris particles. The P1 gate was set to select data representative of *P. gingiavlis*. Data files for each sample were acquired containing a total of 10,000 FSC/SSC P1-gated events (particles). *P. gingivalis* was specifically identified within P1-gated events by FITC fluorescence (with FITC-positive events being selected in the P2 gate) (Figure 1). The attachment of DiD-labelled LL17-32 loaded liposomes with *P. gingivalis* was measured by the increase in DiD fluorescence (detected in the APC channel) of FITC-positive bacteria (P2-gated).

#### 2.2.12. Mammalian Cell Culture

Human telomerase immortalized keratinocytes (TIGKs) derived from the human gingival epithelium were grown to 90% confluence at 37 °C and 5% CO_2_-saturated air in Derma Life Basal culture medium. Passages 8 to 17 were used for all experiments.

#### 2.2.13. Cell Viability Assay

The number of adherent viable cells after treatment was assessed using the MTT assay, which is based on the reaction of a colourless MTT tetrazolium salt with cellular reductases to form purple formazan crystals. TIGK cells (100 µL, 7.5 × 10^5^/mL) were seeded in 96-well flat-bottomed plates for 24 h, washed with PBS, and treated with different concentrations (60–240 µg/mL) of loaded/unloaded liposomes/LL17-32 in basal culture medium for 18 h. Cells were then washed with PBS, and 100 µL of 1 mM MTT was added. The plate was further incubated for 4 h at 37 °C, after which the culture medium was removed, and the resultant formazan crystals were dissolved in 200 µL DMSO. After 15 min incubation at 37 °C, the absorbance was measured at 540 nm using a Varioskan LUX Multimode Microplate Reader (Thermo Fisher, Scientific, Waltham, MA, USA). The relative cell viability with respect to the untreated control was calculated by Equation (5):

(5) $$Relative\;cell\;viability\;(\%)=\frac{OD\;of\;the\;test\;sample}{OD\;of\;control\left(untreated\right)}\cdot 100$$

#### 2.2.14. Cytotoxicity Assay

A lactate dehydrogenase (LDH) assay was used to assess the extent of cell death. Detection of the cytosolic LDH enzyme activity having been released into the culture medium is an indication of plasma membrane disruption and cell death. TIGK cells (100 µL, 7.5 × 10^5^/mL) were seeded in 96-well plates for 24 h, washed with PBS and treated with different concentrations of loaded/unloaded liposomes/LL17-32 for 18 h. Then, 50 μL of culture media was assayed using an LDH Cytotoxicity Assay Kit, as described by the manufacturer. A linked enzymatic reaction in which LDH catalyses the conversion of lactate to pyruvate by nicotinamide adenine dinucleotide (NAD+) reduction to NADH allows for the measurement of extracellular LDH activity. A tetrazolium salt iodonitrotetrazolium (INT) is then reduced by diaphorase using NADH to create a red formazan product that can be detected at 490 nm. The amount of LDH released into the media directly correlates with the level of formazan production. The cytotoxicity was calculated by Equation (6):

(6) $$Cytotoxicity\left(\%\right)=\frac{\left(sample\;value-low\;control\right)}{\left(high\;control-low\;control\right)}\cdot 100$$

where low control determines the LDH activity spontaneously released from the untreated normal cells (spontaneous LDH release); high control determines the maximum releasable LDH activity in the cells which were treated by the lysis solution.

#### 2.2.15. Statistical Analysis

All experiments were carried out in triplicates (*n* = 3). Results were expressed as means ± standard deviation (SD). Comparisons were made by paired Student’s *t*-test analysis using GraphPad Prism version 9.4.1 for Windows (GraphPad Software, San Diego, CA, USA, www.graphpad.com, accessed on 20 January 2019). Differences were significant at *p* < 0.05 throughout this study.

## 3. Results and Discussion

### 3.1. Characterization of Particle Size, Polydispersity and ζ-Potential

**Figure 2:**
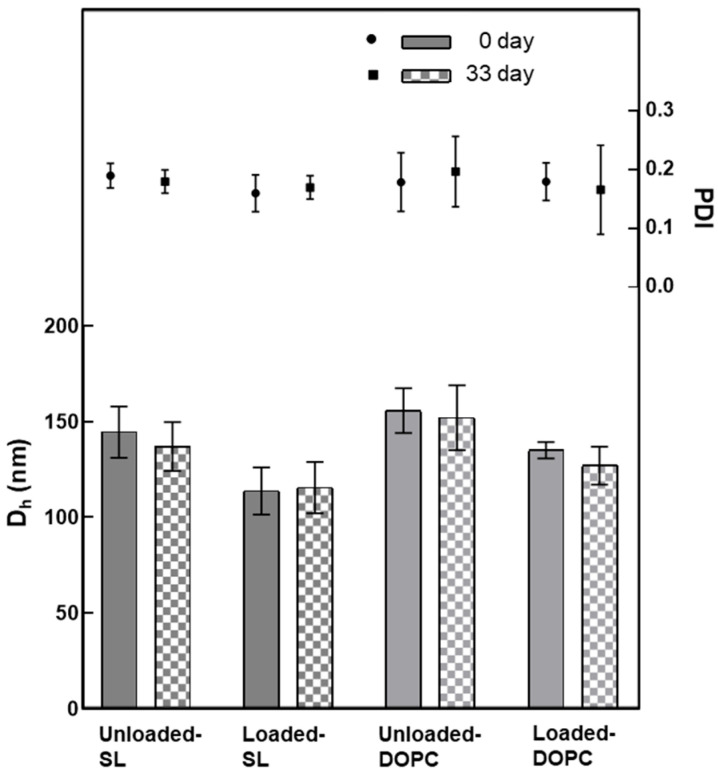
Variation in the average hydrodynamic diameter (*D_h_*) and polydispersity (PDI) of liposomes (SL/DOPC) unloaded and loaded with peptide LL17-32 (600 µg/mL). All formulations were evaluated at 0 and after 33 days of storage at 4 °C (as per legend) (pH = 4.5, 10 mM NaCl, 25 °C, average ± SD, *n* = 3).

To investigate the difference between two types of liposomal formulations (SL and DOPC), DLS-NIBS and ζ-potential measurements were performed immediately after preparation and after 33 days of storage in refrigeration (4 °C). An examination of Figure 2 reveals that, overall, DOPC liposomes were larger in size compared to SL liposomes, regardless of whether they were loaded or unloaded. Additionally, it is notable that upon loading LL17-32 onto both SL and DOPC liposomes, the average particle size decreased by approximately 20 nm relative to their unloaded counterparts. The polydispersity index (PDI) for all formulations consistently remained around 0.2, indicating a narrow and monomodal size distribution. Furthermore, all liposomal formulations demonstrated stability during refrigerated storage for a minimum of 33 days. Several studies have previously explored how the peptide LL-37 affects the properties of liposomes [41,42,43,44]. Several factors contribute to the determination of particle size, including not just the payload and the preparation techniques employed, but also critically the types of lipids used and the cholesterol concentration within the formulation [45]. Cholesterol, as an essential component of liposomes, plays a critical role in their composition and properties. Indeed, studies have shown that cholesterol has a significant impact on the fluidity and permeability of liposomes, primarily through the formation of hydrogen bonding with fatty acids [46]. This interaction enhances the cohesiveness of the liposomal membrane, resulting in increased stability. In a study by Kaddah et al. [47], a gradual increase in the mean size of DPPC vesicles was observed after adding cholesterol at a concentration lower than 30 mol%. The incorporation of cholesterol into the membrane results in the formation of cholesterol-poor and cholesterol-rich domains, which could merge and coalesce into larger vesicles. In another study, Karal et al. [48] examined the effect of elevated cholesterol levels (up to 40 mol%) in giant unilamellar vesicle membranes comprising neutral DOPC, cholesterol, or a mixture of DOPC, cholesterol, and negatively charged 1,2-dioleoyl-sn-glycero-3-phospho-(10-rac-glycerol) (DOPG). In both systems, as the cholesterol concentration increased, the average particle size of the vesicles increased. The impact of cholesterol on the particle size of our DOPC/SL liposomes cannot be directly compared, as all systems were supplemented with ~27 mol% of cholesterol throughout. However, we gained some insight into its role in the membrane fluidity and how it could influence particle size.

As noted, the SL liposomes exhibited smaller sizes than DOPC liposomes by ~20 nm, likely because the SL liposome membrane is more rigid. When large vesicles are forced through the polycarbonate membrane during extrusion, the force can cause them to break apart, and small fragments immediately reassemble into smaller vesicles on the other side of the membrane. However, if the vesicle has a high fluidity, it can be compressed and pass through the polycarbonate membrane pores without being disrupted, thus maintaining its original structure and size when emerging on the other side of the membrane. This interpretation is consistent with our results of membrane fluidity, which will be covered in Section 3.3.

**Figure 3:**
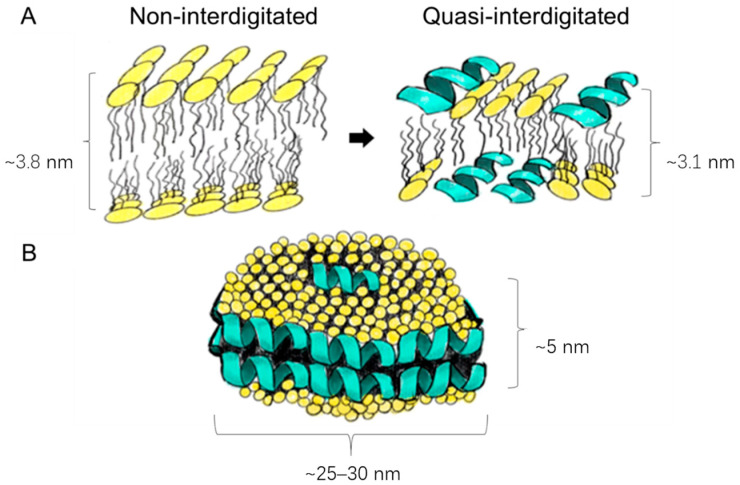
Schematic presentation of the different peptide–lipid arrangements: membrane thinning from a non-interdigitated structure to a quasi-interdigitated (interdigitated) phase (**A**); micellar disks (**B**). Used under CC BY 4.0 [35].

Upon closer examination of Figure 2, it becomes evident that loading LL17-32 onto both SL and DOPC liposomes results in an average particle size reduction of ~20 nm compared to their unloaded counterparts. We suggest that the peptide’s incorporation induces a restructuring of the original lipid membrane configuration. In this context, it is conceivable that a shift occurs from a non-interdigitated structure to a quasi-interdigitated phase (as illustrated in the model in Figure 3A).

Cationic peptides, such as LL-37, are known to be able to penetrate much deeper into the interior of neutral membrane bilayers [49]. If the double diameter of an α-helix is similar in size to the thickness of the hydrophobic core of the membrane, the peptide can position itself within the hydrophobic core, with its hydrophobic side facing the hydrocarbon chains, like the arrangement found in the disk-like micelles (Figure 3B). Although LL-37 orientates itself parallel to the zwitterionic membrane surface with a higher possibility of forming a non-interdigitated structure compared to negatively charged lipids, many other parameters are bound to influence peptide/lipid assemblies such as peptide-to-lipid ratio, types of lipids and peptides, hydration levels of the bilayer, and electrostatic factors [50,51,52,53,54]. While the formation of interdigitated assemblies (Figure 3A) during the preparation or extrusion step could contribute to the reduced size of peptide-loaded liposomes compared to their unloaded counterparts, the primary factor that underpins the size difference in our study is likely to be the variance in membrane fluidity. Specifically, peptide-free liposomes have demonstrated higher membrane fluidity than their loaded counterparts, as detailed in Section 3.3. As already discussed, liposomes with greater fluidity possess the capability to compress and traverse polycarbonate membrane pores without disruption, thus preserving their original size. Conversely, liposomes with more rigid membranes are prone to tearing apart and subsequently reassembling into smaller vesicles.

**Figure 4:**
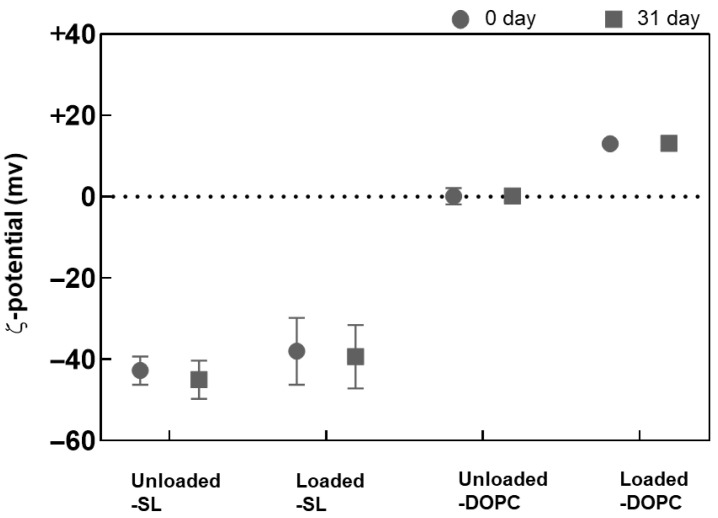
Variation in the ζ-potential of SL/DOPC liposomes unloaded and loaded with peptide LL17-32 (600 µg/mL). All formulations were evaluated at 0 and after 33 days of storage at 4 °C (as per legend) (pH = 4.5, 10 mM NaCl, 25 °C, average ± SD, *n* = 3).

The ζ-potential data corresponding to the SL/DOPC liposomes are shown in Figure 4. A notable observation is the increase in ζ-potential after LL17-32 loading, rising from ~0 mV to ~+15 mV for DOPC liposomes, and from ~−43 mV to ~−35 mV with a comparatively higher standard deviation for SL liposomes. This increase in ζ-potential after LL17-32 loading aligns well with electrostatic principles, as LL17-32 has a isoelectric point of 12, giving rise to a net positive charge that partially neutralizes and binds to phospholipid head groups, including zwitterionic DOPC. Besides electrostatic interactions between anionic phospholipids and cationic AMPs, LL17-32, rich in arginine residues, likely forms bidentate hydrogen bonds between the guanidinium groups of arginine and phosphate, significantly contributing to AMP–membrane interactions. Additionally, lysine residues can form monodentate hydrogen bonds with phosphate groups, further stabilizing the interaction [55,56]. The ζ-potential shift supports LL17-32 binding to the lipid bilayer surface through a combination of electrostatic attraction, hydrogen bonding, and hydrophobic interactions. Consistent with size results, both formulations maintained stable ζ-potential during refrigerated storage for at least 33 days.

### 3.2. Transmission Electron Microscopy (TEM) Imaging Studies

**Figure 5:**
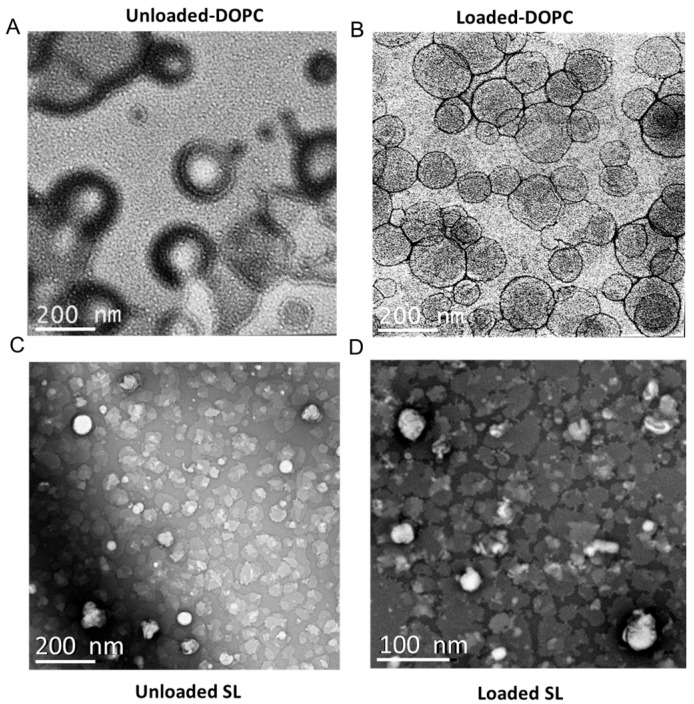
Representative TEM images of liposomal particles: (**A**) LL17-32-unloaded DOPC liposome; (**B**) LL17-32-loaded DOPC liposome; (**C**) LL17-32-unloaded SL liposome; (**D**) LL17-32-loaded SL liposome. Loaded liposomes were loaded with 600 µg/mL LL17-32. Staining was performed with uranyl acetate (1%). Magnification bars are shown on each micrograph.

The size and surface characteristics of liposomes were visualized using transmission electron microscopy (TEM). Both liposome types displayed a spherical morphology, as shown in Figure 5. An interesting aspect of uranyl ions, used as contrast enhancers, is their ability to interact with the phosphate groups present in phospholipids [57], which results in positive stained images. Upon closer examination, it becomes evident that the DOPC liposomes (Figure 5A,B) were larger than the SL liposomes (Figure 5C,D), corroborating the DLS findings (Figure 2). Nevertheless, capturing high-resolution, distinct images of SL liposomes has been problematic. This difficulty is believed to stem from electrostatic repulsion caused by the highly negative charge on the surface of SL liposomes, which likely interferes with their adhesion to the negatively charged carbon grid surface. Such repulsive interactions may prevent the liposomes from firmly attaching to the grid, thereby complicating the acquisition of clear and well-defined electron microscopic images.

### 3.3. Liposome Membrane Fluidity and Structure

**Figure 6:**
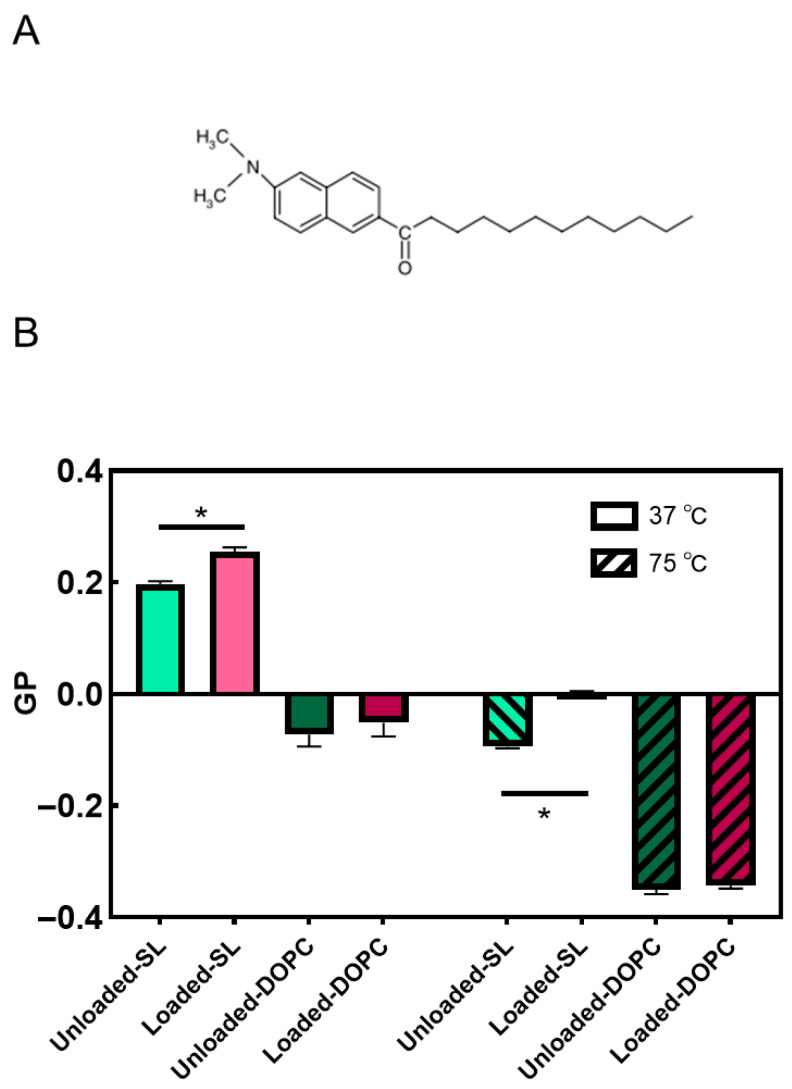
Molecular structure of Laurdan (**A**); effects of LL17-32 on the fluidity of SL/DOPC lipid membrane. GP values calculated for each sample are presented as means ± SD (*n* = 3) at 37 and 75 °C; * *p* < 0.05, Student’s *t*-test. Loaded liposomes were loaded with 600 µg/mL LL17-32 (**B**).

Membrane fluidity represents the extent of molecular disorder and molecular movement within a lipid layer [58]. Membrane fluidity plays a crucial role in the biological function of liposomal systems. It influences liposome–cell membrane interactions, affecting drug delivery efficiency, cellular uptake, and fusion [59,60,61]. As shown in Figure 6B, for DOPC liposomes, negative GP values at both 37 and 75 °C were determined, thus indicating that the membrane was, in both cases, in the liquid crystalline state. DOPC, with an unsaturated chain only, has a well-defined transition temperature of −16.5 °C and therefore is bound to exist in the fluid liquid crystalline state at both temperatures [62]. The fluidity of the lipid membrane is influenced by lipid chain length, degree of saturation, temperature, and cholesterol level [60,63,64,65].

Cholesterol not only has a stiffening effect on saturated lipid membranes but also upon membranes populated by unsaturated lipids such as DOPC. Chakraborty et al. [66] employed a comprehensive approach that integrated neutron spin-echo spectroscopy, solid-state deuterium NMR spectroscopy, and molecular dynamics simulations. The study revealed that cholesterol increases the rigidity of DOPC membranes locally in a manner like that observed in saturated membranes. This effect is attributed to the increase in the bilayer’s packing density. In particular, the addition of cholesterol leads to a noticeable rise in the relative bending rigidity of the membrane as the cholesterol content increases, reaching a value of κ/κ_0_ ~2 for DOPC-Chol membranes containing 30 mol% cholesterol. Furthermore, cholesterol has the effect of reducing the average area per lipid by ~22%. These observations highlight the significant impact of cholesterol on the physical properties of the membrane and its pivotal role in modulating membrane rigidity and lipid packing. Despite the influence of cholesterol on increasing the rigidity and order of DOPC membranes, it is important to note that DOPC remains in the liquid-disordered (L_d_) phase above 10 °C [67]. Also, as the temperature increases to ~75 °C, the membrane exhibits an even higher level of disorder. The decrease in GP values indicates a reduction in the membrane’s overall order and increased fluidity due to the higher temperature-induced disorder. Again, while cholesterol contributes to membrane rigidity and order, it does not induce a phase transition to the liquid-ordered (L_o_) phase in DOPC membranes here. The membrane fluidity and phase behavior of SL liposomes, including effects of LL17-32 loading and temperature-dependent transitions, were comprehensively analyzed in our earlier study supported by SAXS evidence [35]. Briefly, both unloaded and LL17-32-loaded SL liposomes exhibited generalized polarization values indicative of a L_o_ phase at 37 °C. Upon heating to 75 °C, a transition toward the L_d_ phase was observed, consistent with increased membrane fluidity and disappearance of the ordered phase. Moreover, LL17-32 incorporation significantly reduced membrane fluidity at both temperatures.

In summary, our studies revealed that DOPC possesses a generally more fluidic membrane compared to SL liposomes. LL17-32 had a limited impact on the fluidity of the DOPC membrane, likely because DOPC already exists in a liquid crystalline state with high fluidity. However, LL17-32 significantly reduced the fluidity of the SL liposome membrane. This effect may be attributed to the negatively charged SL membranes, which exhibit stronger lipid–peptide interactions driven by electrostatic forces, consequently altering the lipid order within the membranes.

### 3.4. Determination of LL17-32 Association Efficiency (AE) and in Vitro Release in Reduced Transport Fluid (RTF)

The association efficiency (AE) of LL17-32 in both liposomal formulations was determined using reversed-phase HPLC-UV analysis of the free peptide on the ultrafiltrate solution (Figure S1). The results indicated a >~95% AE in all cases, as minimal free LL17-32 peptide was detected. To validate these findings, a positive control involving free LL17-32 loaded into an ultrafiltration centrifuge tube was employed, demonstrating that >~95% of the LL17-32 was recovered. This confirmed the absence of any artifacts introduced by the peptide or the ultrafiltration membrane. The notably high association efficiency observed in the SL liposome can be attributed to the strong electrostatic interactions between the highly positively charged LL17-32 and the negatively charged SL-liposome membranes, thus facilitating peptide–liposome membrane association. Unexpectedly, however, for neutral uncharged DOPC liposomes, the AE was also >~95%. In theory, cationic peptides have a higher binding ability to negatively charged lipid membranes than to neutral ones [68]. Studies compared peptide adsorption to the different charged membrane by null ellipsometry. The higher negative charge of the membranes results in higher adsorption of cationic peptides. Quantitatively, nearly 3-fold higher peptide adsorption is attained for anionic membranes than neutral ones [69,70,71]. However, only peptides dominated by random coil conformation were selected in these studies to gain evidence of the significance of electrostatic interactions, and not α-helical peptides such as LL17-32 used in our work. Other intrinsic features such as length, hydrophobicity, tail moieties, charge, topology and secondary structure all contribute to the ability of peptides to bind to lipid membranes. Also, factors such as peptide-to-lipid ratio, level of cholesterol content, pH, and ionic strength all influence their affinity to liposome membranes [72,73,74,75]. While specific reports on LL17-32 peptide-loaded lipid-based nanostructures are currently lacking, our findings suggest that LL17-32 has potential for effective interaction with both negatively and neutrally charged lipid membranes. However, LL17-32 peptide remained unreleased from any of the systems for up to ~4 h. To confirm this, a positive control was established by loading free LL17-32 peptide into the same dialysis tube, resulting in the recovery of >~90% of the peptide. This control experiment effectively rules out any binding effect between the peptide and the dialysis tube membrane. Considering the absence of LL17-32 release from the system, it is plausible to anticipate that any bactericidal effect of the loaded liposomes on bacteria may not rely on the release of the peptide payload but rather on direct interactions between the liposomal LL17-32 and bacterial cells. However, it should be noted that in vivo conditions differ substantially from the simplified in vitro release assay. Phospholipids such as DOPC and soya lecithin are biodegradable, and enzymatic degradation (e.g., by phospholipases) may destabilize liposomes and facilitate peptide exposure or release, which could contribute to sustained therapeutic delivery.

### 3.5. Colloidal Stability in RTF and in Basal Culture Medium

**Figure 7:**
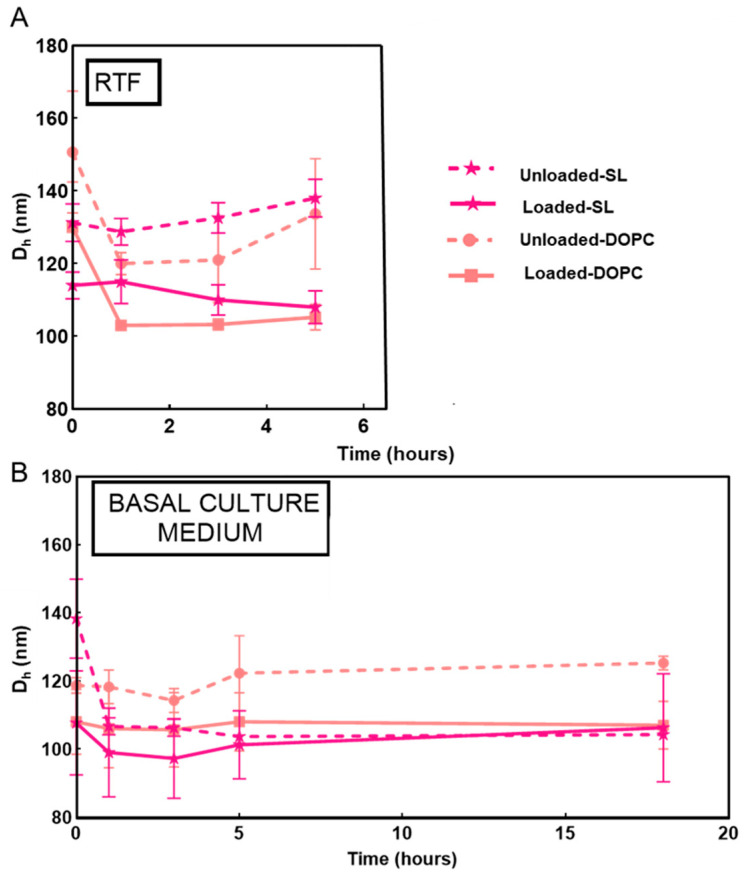
Evolution of the particle size growth of LL17-32-loaded and unloaded SL/DOPC liposomes at varying time intervals incubated in RTF (pH = 7.1) (**A**) and basal culture medium (**B**) at 37 °C. Values are presented as means ± SD (*n* = 3).

The stability of nanoparticles in RTF was investigated from the evolution of the particle size distribution as measured by DLS at varying time intervals up to 4 h at 37 °C. Stability studies on SL and DOPC liposomes revealed that these systems remained stable and showed only a progressive reduction in their size in RTF at early time points (Figure 7). In particular, DOPC liposomes showed a reduction of approximately 20 nm, whereas SL liposomes exhibited less than 10 nm variation in size. These observations agree with previous studies in liposome suspensions (in the absence of added polyelectrolyte) in water–salt media that remained stable, and the vesicles showed only about 20% reduction in size [76]. The observed reduction in liposome size can be explained by the membrane’s impermeability to some ions, which creates osmotic forces and drive water to escape from the liposome’s interior [77].

Given that DOPC/SL liposomes were incubated with TIGK cells in basal culture medium to investigate their cytotoxicity (Section 3.7), their colloidal stability in this medium was also examined (Figure 7). Both LL17-32-loaded and unloaded SL and DOPC liposomes showed similar behavior, remaining stable overall for up to 18 h. A slight decrease in particle size was observed during the first hours of incubation (<20 nm), which can be attributed to osmotic-driven diffusion of water from the liposomal core, as previously suggested for the RTF condition.

### 3.6. Killing Activity on Porphyromonas gingivalis

**Figure 8:**
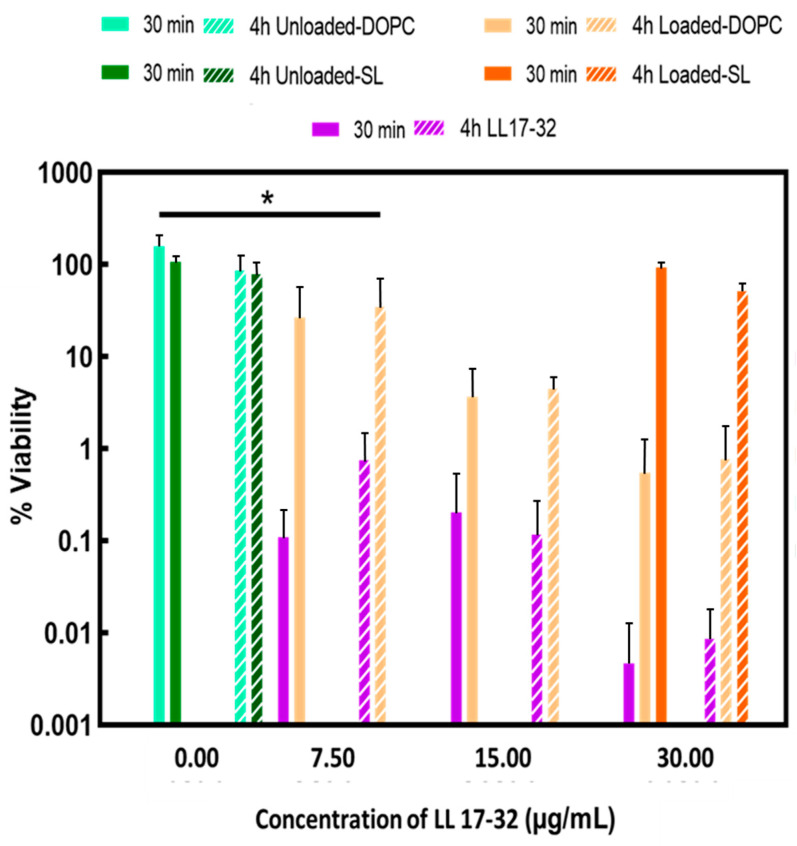
Susceptibility of *P. gingivalis* to peptide and liposome formulations in RTF at 37 °C. Effects of treatment with free-form peptide LL17-32 and LL17-32-loaded/unloaded DOPC/SL liposomes at different concentrations (only the concentration of 30 µg/mL is shown on the graph for LL17-32-loaded SL liposomes) on the bacterial cell viability (after 30 min and 4 h). Cell viability was determined as a percentage of cell viability relative to an untreated control corresponding to 100% viability colony counts (mean ± SD, *n* = 3, * *p* < 0.05, Student’s *t*-test).

The capacity of both LL17-32-loaded and unloaded liposome (DOPC/SL) formulations and free peptide to kill *P. gingivalis* was compared at different peptide concentrations (Figure 8). Both LL17-32-unloaded liposome (DOPC/SL) formulations presented no killing effect on *P. gingivalis* neither after 30 min nor up to 4 h. Unexpectedly, LL17-32-loaded SL formulations did not demonstrate bactericidal effects at peptide concentrations up to 30 µg/mL neither at 30 min nor up to 4 h. By contrast, both the free-form LL17-32 and LL17-32-loaded DOPC liposome did exhibit bacteria-killing effects in a dose-dependent manner in the range 7.5 to 30 µg/mL, with the free peptide being the more potent at equivalent concentrations. Also, this killing effect in both cases was not time-dependent, suggesting rapid peptide-induced killing effects.

Similar results previously reported that both free and liposomal formulated peptides showed similar time–kill profiles against *E. coli* with a rapid bactericidal effect within the first hours of incubation, and a declining bactericidal effect after 6 h [78].

Zwitterionic DOPC liposomes bear positive charges after loading with positively charged peptide LL17-32. We assume this positive charge enables DOPC liposomes to encounter negatively charged bacteria surfaces by electrostatic interaction so that the AMPs can dock on the bacteria membrane, which disrupts the cell membrane and leads to cell death. This hypothesis was further investigated by comparing the adherence between LL17-32-loaded SL/DOPC liposomes and *P. gingivalis* bacteria, which was investigated by flow cytometry as discussed in the next section. Free LL17-32 peptide presented a stronger bactericidal effect against *P. gingivalis* compared to LL17-32-loaded DOPC liposomes. We reason that in LL17-32-loaded DOPC liposomes, the peptide is, to some extent, partitioned on the inner surface of the liposome membrane, where contact with bacteria is not favoured. Boge et al. [78] discovered that after post-loading cubosome with LL-37, the peptide exhibited a reduction in its broad-spectrum antimicrobial activity compared to its free form. LL-37-loaded lipid particles presented decreased activity against Gram-positive *S. aureus*. However, the activity of LL-37-loaded cubosome against Gram-negative bacteria was comparable to that of free LL-37 peptide. The bactericidal abilities of the LL-37-loaded cubosomes are potentially influenced by the variations in bacterial cell walls between Gram-positive and Gram-negative bacteria. For another two peptides, AP114 and DPK060, both unformulated and loaded in cubosome assemblies, similar time–kill profiles were reported [78]. In the case of the method of post-loading cubosome with peptides, the payloads were distributed on the outer surface of the cubosome membrane; therefore, it was unlikely that the contact interaction was compromised in this way. By contrast, in the current work, the peptides were loaded during liposome formation and were located within both inner and outer membrane surfaces. That is probably one of the reasons why free-formed LL17-32 presented stronger killing effects than LL17-32-loaded DOPC liposome. Also, the killing effects are dependent on the nature of the individual type of peptide. This result broadly supports the work of other studies that have demonstrated that a high degree of α-helicity of LL-37 enhances its antibacterial activity [79]. Since the α-helicity of LL-37 increases upon cubosome association, LL-37-loaded cubosomes have shown better antibacterial activity, even though a too-high affinity of the LL-37 peptide to the lipid bilayer membrane might reduce the amount of free peptide available for delivery of its antibacterial activity. The LL17-32 investigated in our study has exhibited a high degree of α-helicity in solution [80]. However, no previous reports of investigations show a structural change of LL17-32 peptide upon interacting with lipid membranes. Also, peptides behave differently against different bacteria strains since bacterial membranes’ composition affects their interaction with cationic peptides [81].

### 3.7. Flow Cytometric Analysis of LL17-32-Loaded Liposomes Binding to P. gingivalis

**Figure 9:**
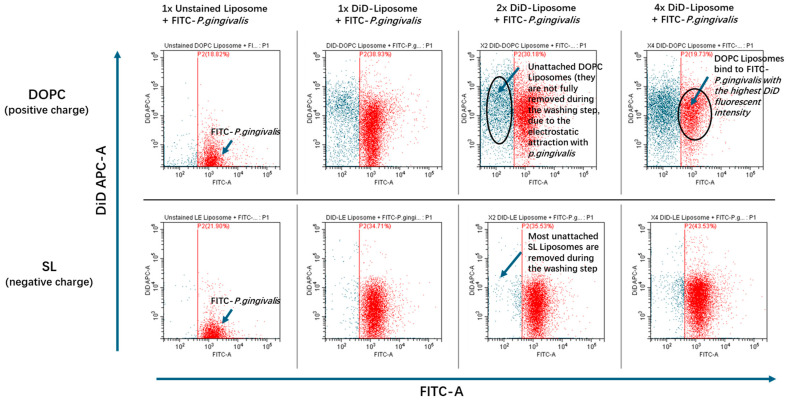
Flow cytometry dot plots of FITC-stained *P. gingivalis* (P2 indicated red) incubated with unstained liposome (Non-DiD) or increasing volumes (1×, 2×, and 4×; corresponding to 10, 20, and 40 µL, respectively) of DiD-stained LL17-32-loaded DOPC/SL liposomes for 30 min at 37 °C with agitation.

To investigate the adherence of LL17-32-loaded SL and DOPC liposomes to *P. gingivalis*, a flow cytometric approach was used to measure the association of fluorescently labelled bacteria and liposome formulations. This enabled us to glean a further understanding of the delivery efficacy of LL17-32 from the different liposomes to bacteria. The results are presented in Figure 9 and Table 1**.** Due to its aliphatic properties, fluorescein perchlorate (DiD) can insert itself into lipid membranes and hence was used to label liposomes during their preparation. Once pre-incorporated into the liposome membrane, the probe is trapped due to its insolubility in aqueous environments so that it cannot diffuse from liposomes to bacteria using the aqueous environment as an intermediate. This implies that any fluorescence transfer from DiD to *P. gingivalis* can only be due to the liposome reaching or attaching to bacteria stained with FITC (*x*-axis). Due to the detection limitations of the flow cytometer, some free liposomes were too small to be recorded; however, the population of *P. gingivalis* was clearly above the threshold of detectable limits. To assess the adherence of LL17-32-loaded DOPC/SL liposomes to *P. gingivalis*, the FITC-stained bacteria were selected (gate P2, Figure 1), and the intensity of their DiD fluorescence (*y*-axis) was measured. The fluorescence data relating to 10,000 FITC-labelled bacteria were collected.

The flow cytometry results showed that for the sample of *P. gingivalis* incubated with LL17-32-loaded DOPC liposome, the geometric mean fluorescence intensity (MFI GeoMean) of DiD increased significantly in a dose-dependent manner (Table 1). By contrast, for the LL17-32-loaded SL liposome formulation, the MFI GeoMean (DiD-A) only increased to around 10% of the intensity gained from LL17-32-loaded DOPC liposome-engaged sample (at the highest liposome concentration of the treated groups). This result suggests that the interaction between positively charged LL17-32-loaded DOPC liposome and *P. gingivalis* was much stronger than that of the negatively charged LL17-32-loaded SL liposome.

The adsorption or binding of liposomes to bacteria may contribute to efficient drug delivery as the intimacy between liposomes and bacteria allows sufficient transfer of the bioactive payload from the lipid layers into the bacterial cell wall. In view of the net negative charge on the surface of most bacteria, the negatively charged SL liposome may bind to bacteria with reduced efficiency due to electrostatic repulsion. On the other hand, the enhanced interaction between positively charged LL17-32-loaded DOPC liposome to bacteria correlated to their enhanced killing efficiency against bacteria, which was shown in the previous results (Figure 8). Similar results were reported on wheat germ agglutinin (WGA)-modified liposomes, which delivered more AMP (temoporfin) to bacteria compared to nonmodified liposomes, since nonmodified liposomes bear a more negative charge [82]. Another study performed by this group integrated a novel AMP (WLBU2) into liposomes. Results showed that more temoporfin was delivered to bacteria by WLBU2-modified liposomes than by unmodified liposomes because the negatively charged unmodified liposomes failed to interact with bacteria due to the electrostatic repulsion [83]. Also, Sugano et al. [84] compared the effect of cationic liposomes and conventional liposomes on interactions with planktonic and biofilm forms of *Streptococcus mutans*. Results showed that liposomes with cationic charges had a higher affinity to bacteria and biofilms than conventional liposomes with negative charges. This study confirmed the previous hypothesis that by comparing adherence between LL17-32-loaded SL/DOPC liposomes to *P. gingivalis*, positively charged DOPC liposomes can be attracted to negatively charged bacteria surface by electrostatic interaction so that the AMPs can interact with the bacterial membrane. These interactions would then disrupt the cell membrane, leading to cell death. By contrast, this interaction is greatly hampered by electrostatic repulsion for negatively charged SL liposomes. This result is in keeping with the limited bacteria-killing effect observed (Figure 8).

### 3.8. Cell Viability and Cytotoxicity

**Figure 10:**
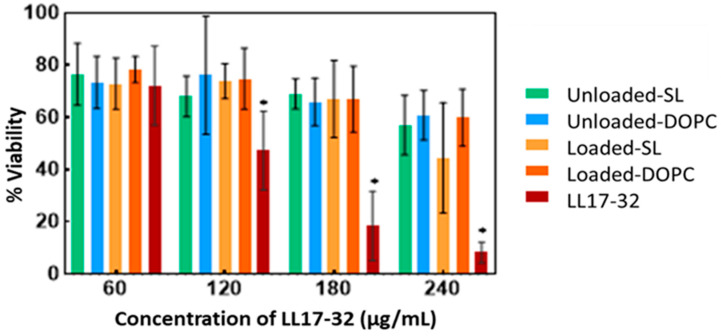
Cell viability (MTT assay) of free LL17-32 peptide, liposomal LL17-32 (Loaded-SL and Loaded-DOPC), and unloaded liposomes (Unloaded-SL and Unloaded-DOPC) against TIGK cells, determined 18 h post-treatment. For unloaded liposomes, data are shown at the same nominal peptide concentrations (60–240 µg/mL) as the loaded formulations to match the added liposome volumes, although they contained 0 µg/mL LL17-32. Cell viability was quantified relative to untreated controls (100% viability). Data represent mean ± SD (*n* = 3); * *p* < 0.05 (Student’s *t*-test).

Understanding the potential cytotoxic effects of the LL17-32 peptide and the liposomal formulation on human cells is essential as an early step preceding pre-clinical studies to anticipate its potential for clinical translation. Therefore, the metabolic activity (as a proxy for cell viability) and cytotoxicity (assessed through cell membrane integrity) of the human gingival keratinocyte cell line (TIGK) were assayed by the well-established MTT and LDH assays, respectively, following treatment with free LL17-32 and liposomal SL and DOPC LL17-32 formulations. The MTT test after 18 h incubation (Figure 10) showed that these mammalian cells treated with free LL17-32 significantly decreased their viability in a dose-dependent manner (*p* < 0.05). Also, free LL17-32 exhibited significantly greater cytotoxicity than SL and DOPC liposomal formulations (*p* < 0.05). The observed variations in cell viability and cytotoxicity were in agreement between both assays (Figure S4). The LDH assay demonstrates larger error bars compared to the MTT assay, reflecting greater inherent variability associated with measuring extracellular enzyme release as a marker of cytotoxicity. This increased variability arises from factors such as spontaneous LDH release from viable cells, differences in cell membrane permeability, efficiency of cell lysis, and background enzymatic activity in the assay medium, all contributing to fluctuations in measured LDH levels. In contrast, the MTT assay measures mitochondrial metabolic activity, which tends to produce more stable and consistent results with lower variability [85]. Ron-Doitch et al. [44] reported that LL-37-loaded liposomes were found to be significantly less cytotoxic than the free peptide form (20–100 μM). However, for liposomal indolicidin, the free drug presented higher or lowered cytotoxicity depending on the different concentrations with no correlation. By comparing two types of liposomes, there was no significant difference in cell viability between loaded or unloaded DOPC- and SL liposome-treated samples. However, both loaded/unloaded DOPC- and SL liposome-treated samples showed slightly decreased cell viability with increased concentration. It should be noted that at high concentrations, treatments of liposomal LL17-32 entailed the addition of a higher volume of applied treatments, reducing the culture media, potentially exposing cells to inadequate culture media and limited nutrients, which could have also amplified the observed cellular death manifesting as drug cytotoxicity. It is well established that liposomes can be internalized by mammalian cells through endocytosis. We hypothesize that the observed reduction in cytotoxicity may be attributed to the LL17-32 attachment on the inner and outer liposome membrane surface, which alters its interaction with TIGK cell membranes. Unlike free LL17-32, which directly disrupts the mammalian cell membrane, liposomal LL17-32 is likely taken up by cells via endocytic pathways, leading to its intracellular degradation rather than immediate membrane disruption. This intracellular processing may contribute to the reduced cytotoxic effects observed. *P. gingivalis* exhibits a dual lifestyle in periodontal sites, residing both within subgingival plaque biofilms and intracellularly within gingival host cells gingival host cells [11]. Given this intracellular persistence, it would be interesting to investigate whether liposomal LL17-32 can be taken up by *P. gingivalis*-infected gingival host cells and effectively eliminate the bacteria within the intracellular environment in future studies. While these current results provided useful first insights into biosafety, further studies are needed to expand the safety evaluation. In particular, assessing immune cell viability and performing hemolysis tests will be important steps to establish a more comprehensive biosafety profile of LL17-32-loaded liposomes.

## 4. Conclusions and Outlooks

AMPs have emerged in recent decades as a large family of natural compounds used by living organisms as part of their natural defence arsenal to fight microbial pathogens. The present study was designed to examine the influence of the two types of liposomal formulations to associate and deliver the AMP LL17-32, which has an established specific bactericidal activity against the dental pathogen *P. gingivalis*. The peptide LL17-32 was loaded into liposomes with high efficiency (>95%), a result ascribed not only to electrostatic interactions but presumably to other interactions, such as hydrophobic interactions, hydrogen bonding, and van der Waals forces, between the peptide and the lipid membrane. This high association efficiency resulted in a negligible release of the payload under in vitro conditions. Peptide LL17-32 significantly decreased the membrane fluidity of high-T_m_ SL liposome but presented a less rigidifying effect on the highly fluidic membrane system (DOPC-liposome). For the biological studies, LL17-32-loaded DOPC liposome exhibited efficient *P. gingivalis*-killing ability compared to the SL liposome, and these results were further supported by investigating the adherence ability of LL17-32-loaded SL/DOPC liposomes to *P. gingivalis* through flow cytometry. The flow cytometry study demonstrated that the interaction between positively charged LL17-32-loaded DOPC liposome and *P. gingivalis* was up to 10 times stronger (at the highest liposome concentration) than the negatively charged LL17-32-loaded SL liposome. Free LL17-32 displayed dose-dependent cytotoxicity against TIGK cells, while liposomal formulations (both SL and DOPC) significantly reduced cytotoxicity without significant differences between groups.

Several liposomal-based commercial products, including DepoDur^®^, AmBisome^®^, Exparel^®^, Onivyde^®^, Marqibo^®^, Doxil^®^, DepoCyt^®^, and Arikayce^®^, are available for intravenous, local injection, or inhalation delivery [61,86]. However, AMP therapeutics account for a very small percentage of all new drugs approved by the FDA. As a result of the limited biological activities in the complex physiological environment, several materials did not perform well in pre-clinical and clinical experiments. Only seven AMPs are currently approved for clinical use and no AMP-based nanomedicines are available for biomedical applications [87]. The results of the present study have implications for understanding the considerations in the initial development stages of liposomes for delivering AMPs in antibacterial applications. Much more work still needs to be carried out; further progress requires a deeper understanding of how peptide-associated nanoparticles behave in complex physiological environments. Because peptides are highly dynamic molecules, their stability and interactions with lipid membranes, proteins, and other biological components need to be systematically evaluated under varying conditions. Future research should also consider that the antimicrobial effects of AMP are not only by direct peptide–bacterial membrane interaction but involve immunomodulation under in vivo conditions [88]. Also, the synergistic effect of other antimicrobial compounds is worthy of consideration. Importantly, given that periodontal disease is driven by complex multi-bacterial biofilms rather than single pathogens, evaluating AMP-loaded liposomes in co-culture models with host cells, in polymicrobial biofilm systems [89], and in animal models of periodontitis will be essential to assess their true translational potential. Developing advanced models and protocols to replicate physiological complexity will be essential for accurately predicting therapeutic performance and guiding the clinical translation of AMP–liposome systems.

## Figures and Tables

**Table 1:** Summary of the geometric mean fluorescence intensity (MFI GeoMean) of the DiD channel for the FITC-positive gated population within each sample. Flow cytometry analysis of FITC-stained *P. gingivalis* incubated with unstained (Non-DiD) and series volumes of DiD-stained LL17-32-loaded DOPC/SL liposomes (1×, 2×, 4×) for 30 min at 37 °C with agitation.

DOPC Liposomes + *P. gingivalis*	MFI GeoMean (DiD-A)	SL Liposomes + *P. gingivalis*	MFI GeoMean (DiD-A)
Unstained DOPC Liposome + FITC-*P. gingivalis*	110 ± 82	Unstained SL Liposome + FITC-*P. gingivalis*	105 ± 72
1× DiD-DOPC Liposome + FITC-*P. gingivalis*	2609 ± 1003	1× DiD-SL Liposome + FITC-*P. gingivalis*	767 ± 559
2× DiD-DOPC Liposome + FITC-*P. gingivalis*	7551 ± 3394	2× DiD-SL Liposome + FITC-*P. gingivalis*	1094 ± 790
4× DiD-DOPC Liposome + FITC-*P. gingivalis*	15,690 ± 9361	4× DiD-SL Liposome + FITC-*P. gingivalis*	1855 ± 1347

## Data Availability

The original contributions presented in this study are included in the article/Supplementary Material. Further inquiries can be directed to the corresponding authors.

## Supplementary Materials

The following supporting information can be downloaded at https://www.mdpi.com/article/10.3390/pharmaceutics17111424/s1, Figure S1: Standard curve to determine the LL17-32 concentration by using reversed-phase HPLC method. Figure S2: Flow cytometry scattering patterns of FITC (0.01–0.2 mg/mL) stained *P. gingivalis*. Figure S3: The MFI geometric mean (GeoMean) of the FITC channel for various concentration FITC labelled *P.gingivalis*. Figure S4: Cytotoxicity (LDH assay) of free LL17-32 peptide, liposomal LL17-32 (Loaded-SL and Loaded-DOPC), and unloaded liposomes (Unloaded-SL and Unloaded-DOPC) against TIGK cells, determined 18 h post-treatment. For unloaded liposomes, data are shown at the same nominal peptide concentrations (60–240 µg/mL) as the loaded formulations to match the added liposome volumes, although they contained 0 µg/mL LL17-32. Cytotoxicity was expressed relative to 10% Triton X-100 (100% toxicity). Data represent mean ± SD (*n* = 3); * *p* < 0.05 (Student’s *t*-test).
