# Supplementary material for: DOPC Liposomal Formulation of Antimicrobial Peptide LL17-32 with Reduced Cytotoxicity: A Promising Carrier Against Porphyromonas gingivalis
Source: Pharmaceutics. 2025 Nov 4;17(11):1424. doi: 10.3390/pharmaceutics17111424 (PMC12655430; doi:10.3390/pharmaceutics17111424)
Supplement: Supplementary file 1 [file pharmaceutics-17-01424-s001.zip › pharmaceutics-3930449-supplementary.pdf]

Supplementary Information

DOPC Liposomal Formulation of Antimicrobial Peptide LL17-32 with Reduced Cytotoxicity: A Promising Carrier against *Porphyromonas gingivalis*

Jinyang Han [1], Josephine Meade[2*] and Francisco M. Goycoolea[1,3,*]

[1] School of Food Science and Nutrition, University of Leeds, Leeds, LS2 9JT, UK; kristinajinyang@gmail.com
[2] School of Dentistry, Oral Biology, University of Leeds, Leeds, LS2 9JT, UK
[3] Department of Cell Biology and Histology, Faculty of Biology, Universidad de Murcia, Murcia, 30100, Spain
* Correspondence: j.l.meade@leeds.ac.uk (J.L.M.); fmartin.goycoolea@um.es (F.M.G.)

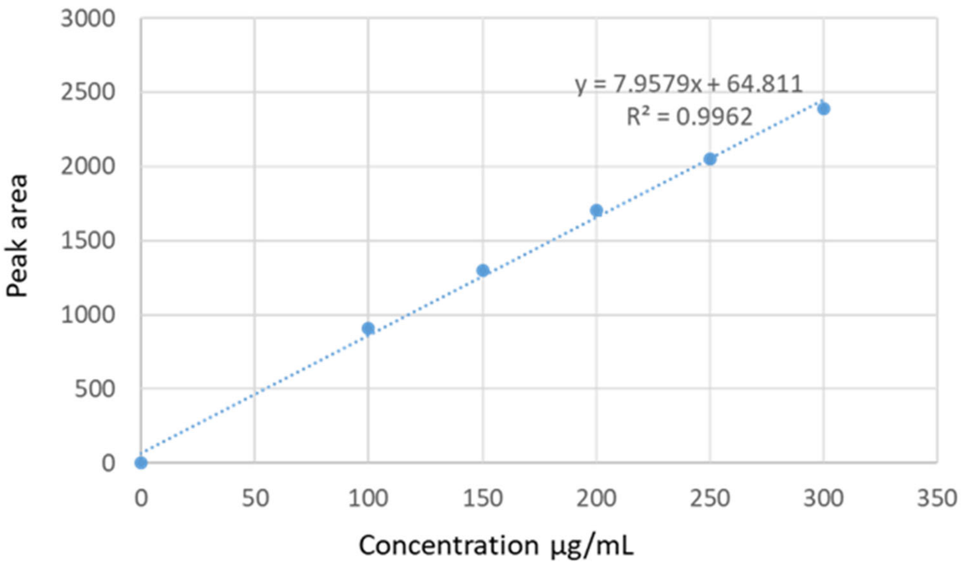

Figure S1. Standard curve for determine the LL17-32 concentration by using reversed-phase HPLC method.

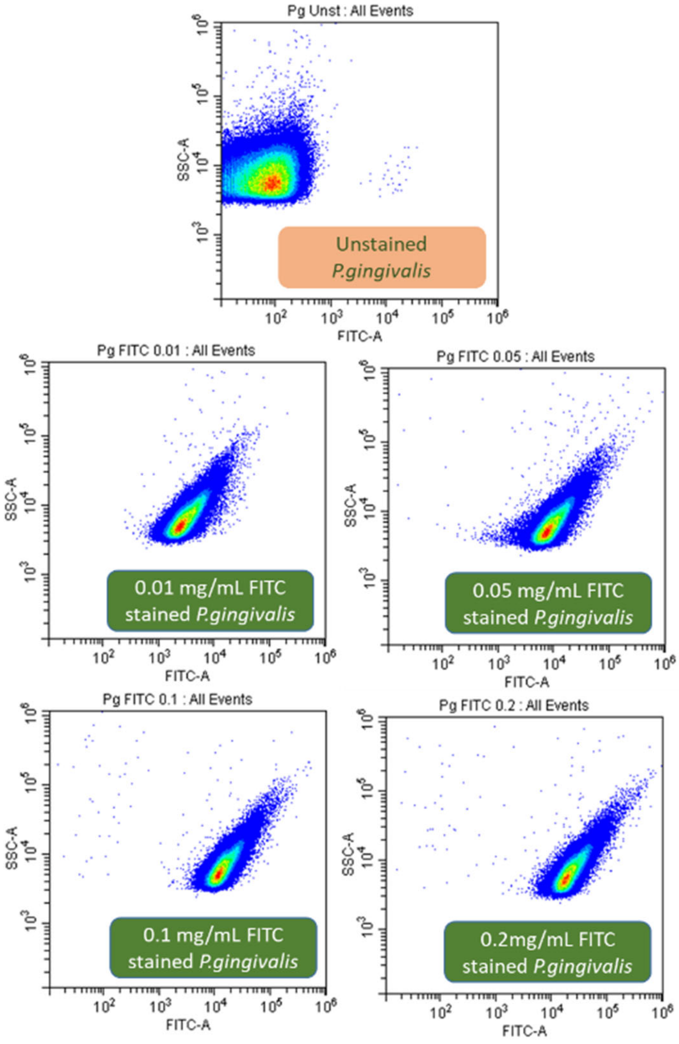

Figure S2. Flow cytometry scattering patterns of FITC (0.01-0.2 mg/mL) stained *P. gingivalis.*

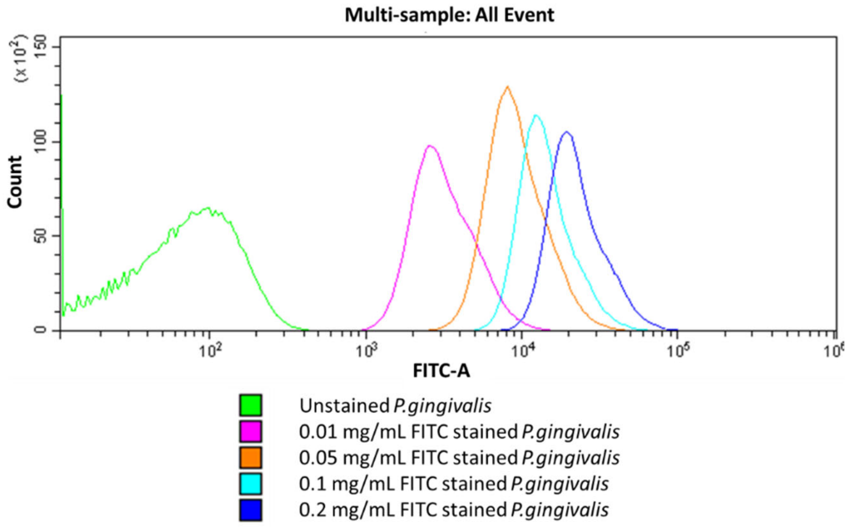

Figure S3. The MFI geometric mean (GeoMean) of the FITC channel for various concentration FITC labelled *P.gingivalis.*

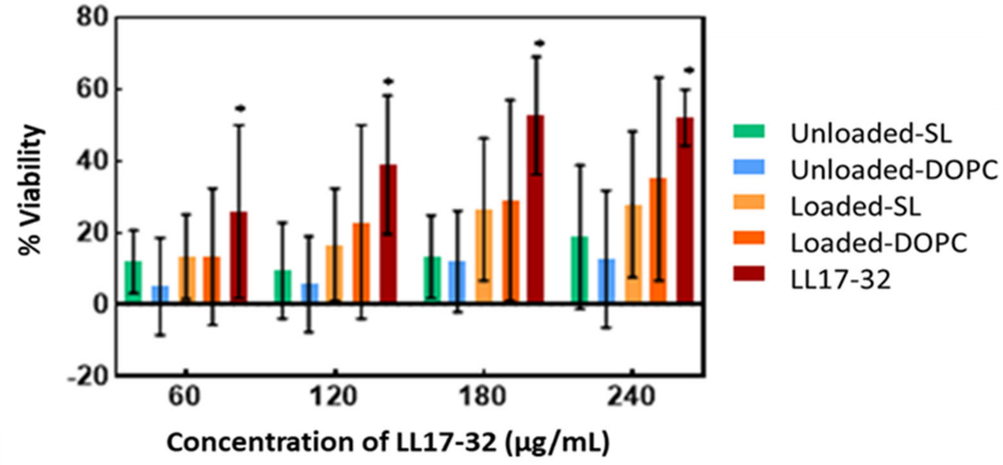

Figure S4. Cytotoxicity (LDH assay) of free LL17-32 peptide, liposomal LL17-32 (Loaded-SL and Loaded-DOPC), and unloaded liposomes (Unloaded-SL and Unloaded-DOPC) against TIGK cells, determined 18 h post-treatment. For unloaded liposomes, data are shown at the same nominal peptide concentrations (60–240 µg/mL) as the loaded formulations to match the added liposome volumes, although they contained 0 µg/mL LL17-32. Cytotoxicity was expressed relative to 10% Triton X-100 (100% toxicity). Data represent mean ± SD (n = 3); *$p < 0.05$ (Student's t-test).
